# Utility of Combining Transcranial Sonography and MIBG Myocardial Scintigraphy to Evaluate Substantia Nigra in Patients with Parkinson’s Disease

**DOI:** 10.3390/brainsci14060524

**Published:** 2024-05-21

**Authors:** Heisuke Mizukami, Makoto Shiraishi, Sakae Hino, Mayumi Kaburagi, Hirofumi Matsumoto, Yuta Hagiwara, Yoshihisa Yamano

**Affiliations:** Division of Neurology, Department of Internal Medicine, St. Marianna University School of Medicine, Kawasaki 216-8511, Japan; h2mizukami@marinna-u.ac.jp (H.M.); shira@marianna-u.ac.jp (M.S.); sakae.hino@marianna-u.ac.jp (S.H.); mayumi.hasegawa@marianna-u.ac.jp (M.K.); hirofumi.matsumoto@marianna-u.ac.jp (H.M.); y2hagiwara@marianna-u.ac.jp (Y.H.)

**Keywords:** Parkinson’s disease, transcranial sonography, dopamine transporter scintigraphy, iodine-123 metaiodobenzylguanidine myocardial scintigraphy, substantia nigra, clinical research

## Abstract

The utility of transcranial sonography (TCS) remains unclarified for the auxiliary diagnosis of Parkinson’s disease (PD). We investigated iodine-123 metaiodobenzylguanidine (MIBG) and TCS during the examination and diagnosis of high-signal-intensity substantia nigra lesion (HSI-SNL) incidence in PD patients previously diagnosed with dopamine transporter scintigraphy (DAT). The subjects were 67 patients with definitively diagnosed PD after DAT evaluation. Patients with midbrain substantia nigra visible during TCS who previously underwent MIBG were analyzed. The SN+ group comprised patients with extensive pathological HSI-SNL of Okawa class III/IV observed during TCS. The MIBG+ group comprised patients with a heart-to-mediastinum ratio of ≤2.2 during MIBG. TCS was performed to divide patients into the SN+ and SN− groups, and patient characteristics and MIBG findings were compared between the groups. PD was definitively diagnosed in 67 patients, among whom midbrain was visualized during TCS in 43 (64.1%) patients and pathological HSI-SNL was observed in 24 (35.8%). The MIBG findings were normal in six patients (27.3%) with HSI-SNL, and abnormal in seven (63.6%) without HSI-SNL. No significant differences were noted by Okawa classification in clinical characteristics based on the presence or absence of HSI-SNL. Multiple patients with normal findings during MIBG may have HSI-SNL. Thus, confirmatory imaging of HSI-SNL with TCS may be useful for diagnosis.

## 1. Introduction

According to the diagnostic criteria for Parkinson’s disease (PD) developed by the International Parkinson and Movement Disorder Society (IPMDS), test methods with specificity exceeding 80% during the differential diagnosis of diseases that present with parkinsonism are said to be the smell identification test and iodine-123 metaiodobenzylguanidine (MIBG) cardiac scintigraphy, whereas normal findings during presynaptic dopaminergic system imaging are considered to be an absolute exclusion criterion [1,2]. However, according to studies of diagnostic precision in cases of PD, approximately 15 to 24% of patients with PD receive an incorrect diagnosis, and the diagnosis of PD itself may be difficult in certain cases [3,4,5]. Transcranial sonography (TCS) has been effectively utilized in Western countries to differentiate between PD and other parkinsonian syndromes [6]. TCS is used to evaluate pathological lesions with high signal intensity within the substantia nigra observed in the midbrain of patients with PD. Compared to other forms of imaging, TCS is useful for PD diagnosis because it is minimally invasive and can be easily performed within a short period of time [7].

It has been reported that DAT scintigraphy and MIBG myocardial scintigraphy are affected by disease duration [8,9,10], but the changes in substantia nigra brightness observed with TCS are not affected by the stage of PD. TCS is a non-invasive test that is better at detecting PD at an asymptomatic or very early stage than myocardial scintigraphy or MIBG myocardial scintigraphy. However, one drawback of TCS is that in many elderly Asian women, it is difficult to observe the midbrain due to their thick temporal bones [11]. It has been shown previously that combining MIBG myocardial scintigraphy and TCS improves the diagnostic accuracy of PD [12], likely due to each test complementing the other’s shortcomings. However, myocardial MIBG scintigraphy, which is widely used in Japan, has not yet become widespread in Europe and the United States due to issues such as insurance coverage and high test costs. In these areas, DAT scintigraphy tends to be used more frequently as a method for the auxiliary diagnosis of PD. Unlike with MIBG myocardial scintigraphy, however, it is difficult to differentiate between PD and other degenerative diseases on DAT scintigraphy [13]. With this background, there are no reports of TCS performed on PD patients who have undergone both DAT scintigraphy and MIBG myocardial scintigraphy with high diagnostic accuracy, and it is possible that existing reports include patients without PD.

In the present study, we aimed to verify the utility of MIBG myocardial scintigraphy and TCS during the examination and diagnosis of high-signal-intensity substantia nigra lesion (HSI-SNL) incidence in patients with PD previously and accurately diagnosed with DAT scintigraphy.

## 2. Materials and Methods

### 2.1. Patients

The subjects in this study were 67 patients who visited a specialized PD outpatient clinic one or more times from 1 April 2020 to 31 March 2021, and were definitively diagnosed as having PD after DAT evaluation. The analysis set comprised subjects in whom the midbrain substantia nigra (MSN) was visible during TCS and who had undergone previous MIBG myocardial scintigraphy. The exclusion criteria were subjects not meeting the diagnostic criteria for PD defined by the IPMDS [1,2], those with poorly visible MSN during TCS, those with a history of cardiac disease, those with a history of diabetes mellitus in whom signs of autonomic neuropathy were observed, those who were taking drugs that affect the results of MIBG myocardial scintigraphy (adrenergic receptor agonists, tricyclic antidepressants), and those in whom head MRI showed findings suggestive of an accumulation of trace metals.

This study was conducted in accordance with the principles of the Declaration of Helsinki. Approval for this study was obtained from the St. Marianna University School of Medicine bioethics committee (approval number 4983: registry study related to the pathological investigation, diagnosis, treatment, and prevention of neurological diseases), and written informed consent was obtained from all subjects included in this study.

### 2.2. Transcranial Sonography

**Figure 1:**
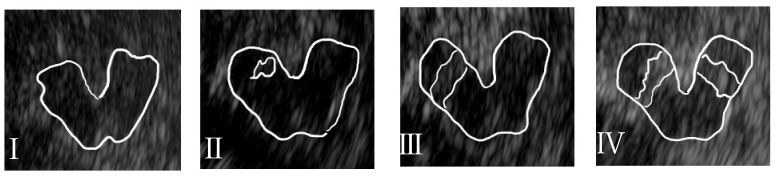
Qualitative classification of midbrain substantia nigra signal intensity. (**I**): None or faint, (**II**): equivocal, (**III**): definite, (**IV**): marked.

TCS was performed using a Xario™ XG ultrasound system (Toshiba, Tokyo, Japan). A sector probe was used to examine the midbrain at a depth of 15 cm via a temporal bone window approach, using the settings of 2.5 MHz and 50–60 dB. Color Doppler imaging was used to identify the transverse plane of the midbrain, with the superior cerebellar artery as the landmark. If possible, the transverse plane image through the midbrain was enlarged and high-signal-intensity regions in the MSN were manually identified. The changes in signal intensity within the MSN were assessed in accordance with the quantitative classification reported by Okawa et al., which is not easily influenced by differences in medical devices or skill levels (Figure 1) [14]. The TCS-based classification includes the following four grades: I: none or faint, II: equivocal, III: definite, and IV: marked. The SN+ group comprised those patients in whom extensive pathological HSI-SNL, corresponding to classes III and IV, were observed. The operators were one physician who was the main observer and one physician who was an assistant. The same physician, a neurologist with extensive clinical experience, was the primary observer in all cases and performed the observations as both the primary observer and as an assistant. Qualitative evaluation of the classification of changes in substantia nigra hyperintensity was performed by the observers and assistants.

### 2.3. DAT Scintigraphy

DAT scintigraphy was performed by first administering 167 MBq [^123^I]ioflupane intravenously, then initiating cranial single-photon emission computed tomography (SPECT) imaging 3 h after administration for a total of 30 min. An Infinia^®^ gamma camera (GE Healthcare, Chicago, IL, USA) was used as the imaging device. Quantitative SPECT image reconstruction was used for analysis. The corpus striatum was set as the region of interest, using a cranial computed tomography image or magnetic resonance image as a reference, to obtain the specific binding ratio (SBR) value as an index of striatal binding ability and for subsequent use for calculation and classification. The age-corrected SBR was defined as being below the 95% confidence interval (CI) [15,16]. Patients in whom the age-corrected SBR was not below this value were excluded from the analysis set as they corresponded to one of the absolute exclusion criteria defined in the IPMDS diagnostic criteria [1,2], namely “the presynaptic dopaminergic neurons are evaluated as normal in functional imaging tests”.

### 2.4. MIBG Scintigraphy

MIBG myocardial scintigraphy was performed by administering 111 MBq (3 mCi) of [^123^I]MIBG via slow intravenous infusion, and then imaging using a gamma camera (Infinia, GE Healthcare) 30 min (early-phase images) and 3 h (late-phase images) later to detect cardiac accumulation. Imaging was performed using the planar method, and the heart-to-mediastinum ratio (H/M ratio) values were corrected based on experiments with a phantom. Subjects with values of 2.2 or less, which were defined as being pathologically decreased, were assigned to the MIBG+ group [17].

### 2.5. Clinical Examination

As the patients with PD were attending a specialized PD outpatient department, the diagnosis of PD and determination of its severity in all subjects were conducted by a highly experienced neurologist. Severity was evaluated using the Hoehn and Yahr scale. Morbidity was defined as the period between the onset of the first motor symptoms and the TCS measurement.

### 2.6. Statistical Analysis

The analysis set comprised patients with PD in whom either MIBG myocardial scintigraphy and/or DAT scintigraphy had been performed. TCS was used to separate patients into the SN+ group and SN− group based on the presence of pathological HSI-SNL. A comparison between the two groups was performed to determine the consistency between the TCS findings and nuclear medicine imaging findings. The data are shown as mean values, standard deviation (SD), and range values. The mean and SD values were determined to perform a comparative investigation of the two groups, and a nonparametric Mann–Whitney U test was used to identify significant differences between continuous variables. The clinical characteristics between the groups were compared using Pearson’s χ^2^ test. The Cochran Q test was used to identify ratios involving three or more groups. Receiver operating characteristic (ROC) curve analysis was used to evaluate the diagnostic value of the MIBG H/M ratio to identify PD patients who were SN+. The optimal cutoff value was calculated by maximizing the sum of sensitivity and specificity. A *p* value of <0.05 was considered significant. EZR ver1.65 was used as the statistical analysis software.

## 3. Results

### 3.1. Midbrain Extraction Rate

**Figure 2:**
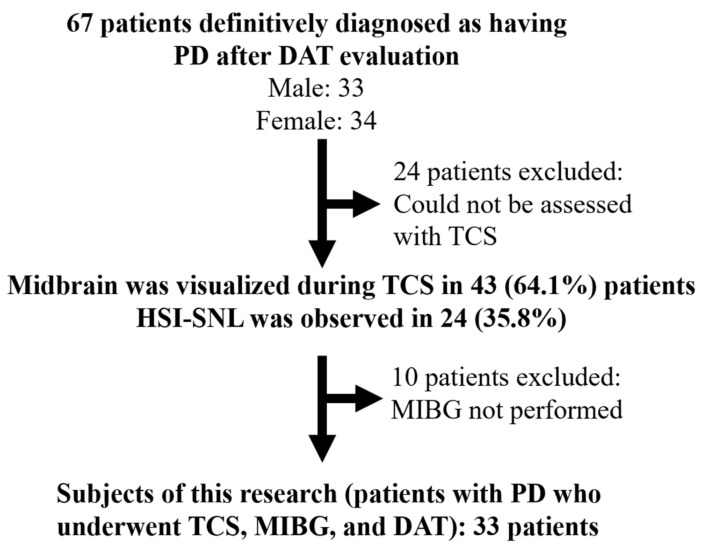
Subject flow in this study.

During the target period, 67 patients (33 men, 34 women) were diagnosed as having PD after visiting the Parkinson’s disease outpatient clinic and having their DAT evaluated. In 43 of the 67 patients (64.1%), the substantia nigra was visualized by TCS, and 24 patients (35.8%) had pathological changes in the substantia nigra. The rate of midbrain visualization was lower in women, 58.8% in 20 of 34 women, compared with 69.7% in 23 of 33 men. The midbrain could not be extracted in 24 of the 67 PD cases because the temporal bone was thick and it was difficult to observe the midbrain. MIBG myocardial scintigraphy was not performed in 10 of the 43 patients in whom visualization by TCS was achieved. Finally, we analyzed 33 PD patients in whom TCS, MIBG myocardial scintigraphy, and DAT scintigraphy were all evaluable (Figure 2).

### 3.2. Characteristics of 33 PD Patients Who Underwent TCS, MIBG Myocardial Scintigraphy, and DAT Scintigraphy

Table 1 shows the patient characteristics of the 33 patients with PD. In terms of sex distribution, men comprised 22 of the patients (66.7%). According to the quantitative substantia nigra classification described by Okawa et al. [14], four patients (12.1%) corresponded to class I, seven (21.2%) to class II, fourteen (42.4%) to class III, and eight (24.2%) to class IV. There were 22 subjects (66.7%) with pathological HSI-SNL (SN+ group). When we compared the clinical characteristics by quantitative substantia nigra classification stage in the 33 patients with PD, there were no significant differences in terms of the early-phase H/M ratio, late-phase H/M ratio, or the washout rate (Table 2). There were no differences in initial symptoms between the different stages. Although a significant difference in levodopa equivalent daily dose was observed between Okawa classification I and II, no difference was observed between SD− (I + II) and SD+ (III + IV).

### 3.3. Hoehn–Yahr Classification and Okawa et al.’s [14] Classification by Sex

Table 3 shows the Hoehn–Yahr classification and Okawa classification by patient sex. There were no obvious differences in the distribution of the Hoehn–Yahr classification and the Okawa classification between the men and women.

### 3.4. Ratio of Abnormal and Normal Findings in MIBG Myocardial Scintigraphy according to TCS Stage

**Figure 3:**
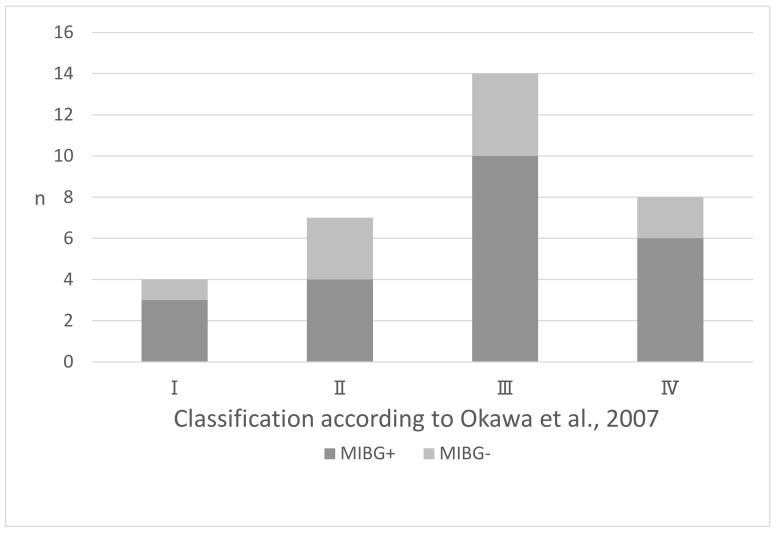
Number of patients with normal and abnormal MIBG myocardial scintigraphy findings by TCS stage among the 33 PD patients who underwent TCS and MIBG and DAT scintigraphy. MIBG+ findings were observed in 22 of the 33 PD patients, whereas MIBG− findings were observed in 10. Among the MIBG+ group, three of four patients corresponded to Okawa class I, four of seven patients to class II, ten of fourteen patients to class III, and six of eight patients to class IV [14]. No differences were observed in terms of ratios in the MIBG+ group among the patients with different Okawa classes.

Abnormal findings were observed during MIBG myocardial scintigraphy (MIBG+ group) in 23 of the 33 patients with PD (69.7%), and 7 of these patients (30.4%) had no pathological high-intensity substantia nigra lesions (SN− group). There were 10 patients with PD among the 33 who had no abnormal findings during MIBG myocardial scintigraphy (MIBG− group), and of these 10 patients, 6 (60%) were also in the SN+ group. Among the MIBG+ group, three of four patients corresponded to Okawa class I, four of seven to class II, ten of fourteen to class III, and six of eight to class IV (Figure 3). No differences were observed in terms of the H/M ratios in the MIBG+ group among the patients in the different Okawa classes, nor were there significant differences in patient characteristics between the SN+ and SN− groups. Meanwhile, the period between onset and imaging showing abnormal MIBG myocardial scintigraphy findings was non-significantly longer in the SN− group than in the SN+ group (3.18 ± 4.53 vs. 2.36 ± 1.76 years; *p* = 0.459) (Table 4).

### 3.5. ROC Curve for SN+ and MIBG H/M Early-Phase Ratio

**Figure 4:**
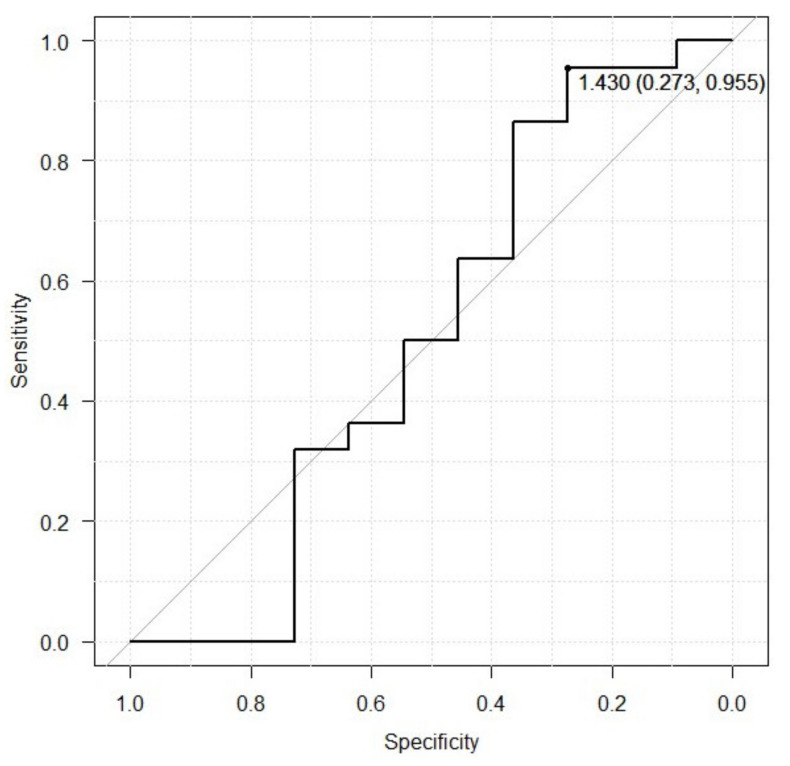
ROC curve for SN+ and MIBG H/M early-phase ratio. The area under the ROC curve of the MIBG H/M early-phase ratio value for identifying SN+ was 0.508 (95% CI, 0.258–0.759). The combination of sensitivity and specificity was maximized when the cutoff value was set to an H/M ratio of 1.43, with a sensitivity of 27.3% and specificity of 95.5% for predicting SN+.

ROC curve analysis was used to evaluate the diagnostic value of the MIBG H/M ratio to identify PD that was SN+. The area under the ROC of the MIBG H/M early-phase ratio value for identifying SN+ was 0.508 (95% CI, 0.258–0.759) (Figure 4). The combination of sensitivity and specificity was maximized when the cutoff value was set to an H/M ratio of 1.43, with a sensitivity of 27.3% and specificity of 95.5% for predicting SN+.

## 4. Discussion

In the present study, we investigated the utility of MIBG myocardial scintigraphy and TCS in patients with PD previously diagnosed with DAT scintigraphy. The results showed that there were six SN+ patients (60%) among the ten MIBG− patients, and seven MIBG+ patients (63.6%) among the eleven SN− patients. This suggests that the addition of TCS to other investigations can act to increase diagnostic precision.

### 4.1. Utility of Combining TCS and MIBG Myocardial Scintigraphy

Among the study patients, 60% (6/10) had visible HSI-SNL during TCS despite being MIBG−, whereas 63.6% (7/11) had no visible HSI-SNL during TCS despite being MIBG+. TCS evaluates deficits in the substantia nigra, whereas MIBG myocardial scintigraphy evaluates postsynaptic cardiac parasympathetic neuron degeneration. It is therefore presumed that the results of these two investigations will not necessarily be consistent, suggesting that there are various forms of PD lesion progression. HSI-SNL also does not necessarily correlate to the clinical PD stage; so, the addition of TCS to the investigation of patients whose MIBG myocardial scintigraphy results were negative offers an excellent chance to screen for PD that is either asymptomatic or at an extremely early stage. Meanwhile, despite the fact that MIBG myocardial scintigraphy may reveal no abnormalities in the early stages of the disease [18], it exhibits high diagnostic sensitivity and specificity and is excellent for differentiating between PD and other degenerative disorders or essential tremors [18]. The fact that adding TCS to MIBG myocardial scintigraphy improves the diagnostic accuracy for PD has already been reported in previous research [12]. The results of the present study suggest that TCS exerts particularly beneficial effects on diagnostic accuracy in suspected cases of PD with normal MIBG myocardial scintigraphy findings. We believe that TCS is also appropriately indicated for patients with suspected degenerative diseases of the substantia nigra, including PD, but who are in the early stage of disease onset, during which it is difficult to distinguish PD from other diseases by MIBG myocardial scintigraphy, or patients with suspected PD who are in the late stage of disease onset but do not show abnormal values on MIBG myocardial scintigraphy.

### 4.2. Incidence of HSI-SNL in Patients with PD

The incidence of PD among those in whom HSI-SNL was observed in the present study was 66.7% (Table 2). The incidence of HSI-SNL in patients with PD has been reported to be 90% or more [19,20,21], and we were surprised to note that the incidence of HSI-SNL was lower in our study. The first reason for this difference could be the impact of age on HSI-SNL. Generally, irrespective of age, the rate of HSI-SNL in healthy adults remains fixed at approximately 10% [21,22], but there is lack of consensus regarding whether this percentage increases with age [23]. The second reason for this difference could be potentially different mechanisms for the effects of PD and age on HSI-SNL. It is presumed that HSI-SNLs due to PD are caused by increased iron levels and the involvement of microglial activity, whereas those due to aging are caused by the accumulation of micro-minerals other than iron. Differentiation is said to be difficult in cases in which there are no differences between the two after searching for TCS findings [19]. The third reason for this difference could be the presence of diseases frequently misdiagnosed as PD. The clinical features of early PD closely resemble those of other neurodegenerative disorders, such as the parkinsonian variant of progressive supranuclear palsy or multiple system atrophy; so, previous reports have suggested that approximately 15 to 24% of patients with PD may have been incorrectly diagnosed [3,4,5]. Considering the above reasons, we presume that there may be a higher-than-expected number of other neurodegenerative disorders and his-SNL due to aging in the previous reports. Furthermore, in the present study, there were no obvious differences between the SN+ and SN− patients in terms of the H/M ratios on MIBG myocardial scintigraphy or the mean SBR observed during DAT scintigraphy (Table 4). Some reports have suggested that the SBR values and H/M ratios decrease as mobility increases [8,9,10]. By contrast, there are also reports hypothesizing that TCS findings reflect a loss of the substantia nigra [24] and that changes in the substantia nigra are not limited to those due to the disease state or morbidity [25]. We presume that due to the differences in the nature of each of these tests, the addition of TCS to DAT scintigraphy and MIBG myocardial scintigraphy will provide useful diagnostic information. In this study, although we investigated the relationship between TCS and MIBG testing using an ROC curve, the AUC was low at 0.508, and the relationship was also considered to be low (Figure 4).

### 4.3. Differences between Men and Women in Changes in Substantia Nigra Hyperintensity in PD

Generally, men are considered to be at higher risk of developing PD, and it has been reported that this difference increases with age. The reasons for sex influencing the onset of PD include the rate of smoking and the neuroprotective effect of estrogen, but to date, this has not been clarified. Although not statistically significant in the present study, the frequency of SN+ tended to be higher in men (Table 3, *p* = 0.0071). A large-scale cross-sectional study investigating the frequency of SN+ in PD reported that SN+ was significantly associated with men, and our study is consistent with that result [26]. As SN+ reflects the vulnerability of the substantia nigra, it is not difficult to imagine that healthy people with SN+ could develop PD later in life. It is possible (hypothetically) that the difference in the frequency of SN+ between men and women influences the difference in the risk of developing PD and the incidence rate in the elderly.

### 4.4. Limitations

This study has several limitations. First, this study was conducted at a single facility with a small number of patients. Second, problems with TCS include the use of qualitative classifications and operator bias. There are no international standards to define changes in substantia nigra hyperintensity, and there are many reports indicating that a change is detected when the change in the area of substantia nigra hyperintensity is of 0.20 cm^2^ or more [27]. However, because TCS results vary widely depending on the observer [28,29], we used a qualitative classification method that is less influenced by the observer and the technical skills of the operator. Third, although this study targeted patients diagnosed as having PD based on DAT scintigraphy, the diagnosis of PD lacked confirmation by autopsy, and it cannot be ruled out that some patients who did not have PD were included. Specifically, this includes patients with other genetic diseases.

To benefit from the use of TCS in the future, we will need to develop more simplistic devices with high detection accuracy and correlate their findings with other diagnostic biomarkers.

## 5. Conclusions

In some instances, patients with normal MIBG myocardial scintigraphy findings may present with HSI-SNL, and conversely, patients without HSI-SNL may present with abnormal MIBG myocardial scintigraphy findings. It is assumed that TCS provides diagnostically useful information independently of MIBG myocardial scintigraphy. In addition, the low frequency of SN+ in this study may suggest that the frequency of SN+ is not as high as previously reported in correctly diagnosed PD.

## Figures and Tables

**Table 1:** Characteristics of the 33 patients with PD.

Characteristic	Value
Male, *n* (%)	22 (66.7)
Age, years	73.1 ± 9.6
Onset age, years	67.5 ± 0.2
Disease duration, years	5.6 ± 3.2
LEDD, mg	589.9 ± 285.3
Initial symptom, *n* (%)	
Tremor	18 (54.5)
Rigidity	7 (21.2)
Bradykinesia	8 (24.2)
Hoehn–Yahr stage, *n* (%)	
I	1 (3.0)
II	7 (21.2)
III	16 (48.5)
IV	9 (27.3)
V	0 (0)
Substantia nigra signal intensity by Okawa et al. [14] classification, *n* (%)	
I	4 (12.1)
II	7 (21.2%)
III	14 (42.4)
IV	8 (24.2%)
III + IV	22 (66.7%)
MIBG myocardial scintigraphy	
Period from onset until imaging, years	3.27 ± 1.97
Early-phase H/M ratio	2.01 ± 0.57
Late-phase H/M ratio	1.65 ± 0.66
Washout ratio, %	63.2 ± 24.0
DAT scintigraphy	
Mean SBR	2.31 ± 1.01
AI, %	23.18 ± 31.58

PD = Parkinson’s disease, LEDD = levodopa equivalent daily dose, MIBG = metaiodobenzylguanidine, H/M = heart-to-mediastinum ratio, DAT = dopamine transporter, SBR = specific binding ratio, AI = asymmetry index. Values are means ± SD.

**Table 2:** Clinical characteristics by quantitative substantia nigra classification stage of the 33 patients with PD who underwent TCS and MIBG and DAT scintigraphy.

	I	II	III	IV	*p* Value
	*n* = 4	*n* = 7	*n* = 14	*n* = 8	
	SN− *n* = 11, 33.3%		SN+ *n* = 22, 66.7%		
Age, years	72.75 ± 9.74	76.85 ± 4.33	72.85 ± 10.33	70.62 ± 12.15	0.678
Onset age, years	64.75 ± 9.53	71.00 ± 7.23	68.07 ± 10.82	64.85 ± 12.27	0.661
Disease duration, years	8.00 ± 1.63	5.86 ± 5.98	4.79 ± 1.97	5.75 ± 2.05	0.385
LEDD, mg	895 ± 414	377 ± 200	656 ± 261	507 ± 151	0.012 *
	565 ± 379		602 ± 235		0.606
Initial symptom, *n*					0.761 *
Tremor	2	4	7	5	
Rigidity	1	0	4	2	
Bradykinesia	1	3	3	1	
MIBG myocardial scintigraphy					
Period from onset until imaging, years	1.50 ± 1.91	4.14 ± 5.43	1.71 ± 1.32	3.50 ± 1.92	0.213
Early-phase H/M ratio	1.82 ± 0.39	2.15 ± 0.86	2.04 ± 0.49	1.94 ± 0.49	0.801
Late-phase H/M ratio	1.42 ± 0.46	1.92 ± 0.91	1.57 ± 0.41	1.67 ± 0.84	0.605
Washout rate, %	72.08 ± 29.5	55.70 ± 27.3	61.98 ± 17.8	67.65 ± 30.5	0.695
DAT scintigraphy					
Mean SBR	2.65 ± 1.66	2.30 ± 0.91	2.05 ± 0.90	2.60 ± 1.00	0.595
AI, %	51.50 ± 73.1	11.40 ± 9.1	25.00 ± 26.4	16.28 ± 16.0	0.209

PD = Parkinson’s disease, TCS = transcranial sonography, MIBG = metaiodobenzylguanidine, DAT = dopamine transporter, SN = substantia nigra, LEDD = levodopa equivalent daily dose, H/M = heart-to-mediastinum ratio, SBR = specific binding ratio, AI = asymmetry index. * Fisher’s exact test. Values are means ± SD.

**Table 3:** Hoehn–Yahr classification and Okawa et al. [14] classification by sex and substantia nigra signal intensity by Okawa et al. classification [14].

	Male	Female	*p* Value
	N = 22	N = 11	
Age, years	72.2 ± 10.9	75.1 ± 6.6	0.422
Onset age, years	66.9 ± 11.7	68.8 ± 6.7	0.612
Disease duration, years	5.3 ± 2.5	6.3 ± 4.4	0.434
Period from onset until imaging, years	2.5 ± 1.8	2.9 ± 4.6	0.712
LEDD, mg	630.8 ± 307.3	508.2 ± 226.2	0.251
MIBG myocardial scintigraphy			
Early-phase H/M ratio	2.02 ± 0.54	2.00 ± 0.65	0.953
Late-phase H/M ratio	1.64 ± 0.62	1.68 ± 0.75	0.853
Washout rate, %	63.0 ± 23.2	63.8 ± 26.9	0.923
DAT scintigraphy			
Mean SBR	2.34 ± 0.99	2.23 ± 1.10	0.776
AI, %	21.6 ± 23.0	25.8 ± 45.5	0.739
Initial symptom, *n*			0.165 *
Tremor	14	4	
Rigidity	5	2	
Bradykinesia	3	5	
Hoehn–Yahr stage, *n*			0.136 *
I	0	1	
II	6	1	
III	12	4	
IV	4	5	
V	0	0	
Substantia nigra signal intensity by Okawa et al. [14] classification, *n*			0.108 *
I	3	1	
II	2	5	
III	10	4	
IV	7	1	
III + IV	17	5	0.071

LEDD = levodopa equivalent daily dose. * Fisher’s exact test. Values are means ± SD.

**Table 4:** Ratio of those with versus without HSI-SNL among the 33 patients with PD who underwent TCS, MIBG, and DAT scintigraphy.

HSI-SNL	SN+	SN−	*p* Value
	*n* = 22	*n* = 11	
Male, *n* (%)	17 (77.2)	5 (45.5)	0.117 *
Age, years	72.04 ± 10.79	75.36 ± 6.63	0.359
Onset age, years	66.90 ± 11.19	68.72 ± 8.28	0.637
Disease duration, years	6.63 ± 4.84	5.13 ± 2.00	0.215
LEDD	565 ± 379	602 ± 235	0.606
MIBG myocardial scintigraphy			
Period from onset until imaging, years	2.36 ± 1.76	3.18 ± 4.53	0.459
Early-phase H/M ratio	2.00 ± 0.48	2.03 ± 0.73	0.881
Late-phase H/M ratio	1.61 ± 0.59	1.74 ± 0.79	0.575
Washout rate, %	64.04 ± 22.6	61.65 ± 27.9	0.793
DAT scintigraphy			
Mean SBR	2.25 ± 0.95	2.42 ± 1.16	0.644
AI, %	21.83 ± 23.1	25.89 ± 45.3	0.734

HSI-SNL = high-signal-intensity substantia nigra lesion, PD = Parkinson’s disease, TCS = transcranial sonography, MIBG = metaiodobenzylguanidine, DAT = dopamine transporter, SN = substantia nigra, LEDD = levodopa equivalent daily dose, H/M = heart-to-mediastinum ratio, SBR = specific binding ratio, AI = asymmetry index. * χ^2^ test. Values are means ± SD.

## Data Availability

The data are available upon request. The data are not publicly available due to their containing information that could compromise the privacy of the study participants.

## References

[1] Postuma R.B., Berg D., Stern M., Poewe W., Olanow C.W., Oertel W., Obeso J., Marek K., Litvan I., Lang A.E. (2015). MDS clinical diagnostic criteria for Parkinson’s disease. Mov. Disord..

[2] Berg D., Adler C.H., Bloem B.R., Chan P., Gasser T., Goetz C.G., Halliday G., Lang A.E., Lewis S., Li Y. (2018). Movement disorder society criteria for clinically established early Parkinson’s disease. Mov. Disord..

[3] Tolosa E., Garrido A., Scholz S.W., Poewe W. (2021). Challenges in the diagnosis of Parkinson’s disease. Lancet Neurol..

[4] Schrag A., Ben-Shlomo Y., Quinn N. (2002). How valid is the clinical diagnosis of Parkinson’s disease in the community?. J. Neurol. Neurosurg. Psychiatry.

[5] Hughes A.J., Daniel S.E., Kilford L., Lees A.J. (1992). Accuracy of clinical diagnosis of idiopathic Parkinson’s disease: A clinico-pathological study of 100 cases. J. Neurol. Neurosurg. Psychiatry.

[6] Vlaar A.M., Bouwmans A.E., van Kroonenburgh M.J., Mess W.H., Tromp S.C., Wuisman P.G., Kessels A.G., Winogrodzka A., Weber W.E. (2007). Protocol of a prospective study on the diagnostic value of transcranial duplex scanning of the substantia nigra in patients with parkinsonian symptoms. BMC Neurol..

[7] Becker G., Seufert J., Bogdahn U., Reichmann H., Reiners K. (1995). Degeneration of substantia nigra in chronic Parkinson’s disease visualized by transcranial color-coded real-time sonography. Neurology.

[8] Satoh A., Serita T., Seto M., Tomita I., Satoh H., Iwanaga K., Takashima H., Tsujihata M. (1999). Loss of ^123^I-MIBG uptake by the heart in Parkinson’s disease: Assessment of cardiac sympathetic denervation and diagnostic value. J. Nucl. Med..

[9] Benamer H.T., Patterson J., Wyper D.J., Hadley D.M., Macphee G.J., Grosset D.G. (2000). Correlation of Parkinson’s disease severity and duration with ^123^I-FP-CIT SPECT striatal uptake. Mov. Disord..

[10] Lavalaye J., Booij J., Reneman L., Habraken J.B., van Royen E.A. (2000). Effect of age and gender on dopamine transporter imaging with [123I]FP-CIT SPET in healthy volunteers. Eur. J. Nucl. Med..

[11] Kajimoto Y., Miwa H., Okawa-Izawa M., Hironishi M., Kondo T. (2009). Transcranial sonography of the substantia nigra and MIBG myocardial scintigraphy: Complementary role in the diagnosis of Parkinson’s disease. Park. Relat. Disord..

[12] Behnke S., Hellwig D., Bürmann J., Runkel A., Farmakis G., Kirsch C.M., Fassbender K., Becker G., Dillmann U., Spiegel J. (2013). Evaluation of transcranial sonographic findings and MIBG cardiac scintigraphy in the diagnosis of idiopathic Parkinson’s disease. Park. Relat. Disord..

[13] Vasilios C.C., Michail S., George P., Maria C., Leonidas S., Elisabeth K. (2023). Dopamine transporter SPECT imaging in Parkinson’s disease and atypical Parkinsonism: A study of 137 patients. Neurol. Sci..

[14] Okawa M., Miwa H., Kajimoto Y., Hama K., Morita S., Nakanishi I., Kondo T. (2007). Transcranial sonography of the substantia nigra in Japanese patients with Parkinson’s disease or atypical parkinsonism: Clinical potential and limitations. Intern. Med..

[15] Booij J., Tissingh G., Boer G.J., Speelman J.D., Stoof J.C., Janssen A.G., Wolters E.C., van Royen E.A. (1997). [123I]FP-CIT SPECT shows a pronounced decline of striatal dopamine transporter labelling in early and advanced Parkinson’s disease. J. Neurol. Neurosurg. Psychiatry.

[16] Plotkin M., Amthauer H., Klaffke S., Kühn A., Lüdemann L., Arnold G., Wernecke K.D., Kupsch A., Felix R., Venz S. (2005). Combined ^123^I-FP-CIT and ^123^I-IBZM SPECT for the diagnosis of parkinsonian syndromes: Study on 72 patients. J. Neural Transm..

[17] Orimo S., Ozawa E., Nakade S., Sugimoto T., Mizusawa H. (1999). (123)I-metaiodobenzylguanidine myocardial scintigraphy in Parkinson’s disease. J. Neurol. Neurosurg. Psychiatry.

[18] Orimo S., Suzuki M., Inaba A., Mizusawa H. (2012). ^123^I-MIBG myocardial scintigraphy for differentiating Parkinson’s disease from other neurodegenerative parkinsonism: A systematic review and meta-analysis. Park. Relat. Disord..

[19] Walter U. (2009). Transcranial brain sonography findings in Parkinson’s disease: Implications for pathogenesis, early diagnosis and therapy. Expert Rev. Neurother..

[20] Walter U., Dressler D., Probst T., Wolters A., Abu-Mugheisib M., Wittstock M., Benecke R. (2007). Transcranial brain sonography findings in discriminating between parkinsonism and idiopathic Parkinson disease. Arch. Neurol..

[21] Berg D., Becker G., Zeiler B., Tucha O., Hofmann E., Preier M., Benz P., Jost W., Reiners K., Lange K.W. (1999). Vulnerability of the nigrostriatal system as detected by transcranial ultrasound. Neurology.

[22] Berg D., Siefker C., Ruprecht-Dörfler P., Becker G. (2001). Relationship of substantia nigra echogenicity and motor function in elderly subjects. Neurology.

[23] Behnke S., Double K.L., Duma S., Broe G.A., Guenther V., Becker G., Halliday G.M. (2007). Substantia nigra echomorphology in the healthy very old: Correlation with motor slowing. Neuroimage.

[24] Zecca L., Berg D., Arzberger T., Ruprecht P., Rausch W.D., Musicco M., Tampellini D., Riederer P., Gerlach M., Becker G. (2005). In vivo detection of iron and neuromelanin by transcranial sonography: A new approach for early detection of substantia nigra damage. Mov. Disord..

[25] Berg D., Merz B., Reiners K., Naumann M., Becker G. (2005). Five-year follow-up study of hyperechogenicity of the substantia nigra in Parkinson’s disease. Mov. Disord..

[26] Schweitzer K.J., Behnke S., Liepelt I., Wolf B., Grosser C., Godau J., Gaenslen A., Bruessel T., Wendt A., Abel F. (2007). Cross-sectional study discloses a positive family history for Parkinson’s disease and male gender as epidemiological risk factors for substantia nigra hyperechogenicity. Neural Transm..

[27] Walter U., Behnke S., Eyding J., Niehaus L., Postert T., Seidel G., Berg D. (2007). Transcranial brain parenchyma sonography in movement disorders: State of the art. Ultrasound Med. Biol..

[28] Gaenslen A., Unmuth B., Godau J., Liepelt I., Santo A.D., Johanna K.S., Gasser T., Machulla H.J., Reimold H., Marek K. (2008). The specificity and sensitivity of transcranial ultrasound in the differential diagnosis of Parkinson’s disease: A prospective blinded study. Lancet Neurol..

[29] Loo S., Walter U., Behnke S., Chen S.D. (2010). Reproducibility and diagnostic accuracy of substantia nigra sonography for the diagnosis of Parkinson’s disease. J. Neurol. Neurosurg. Psychiatry.

